# Supplementary material for: Iron-sulfur clusters in SARS-CoV-2 exoribonuclease and methyltransferase complexes: relevance for viral genome proofreading and capping
Source: Nat Commun. 2025 Aug 15;16:7585. doi: 10.1038/s41467-025-62832-5 (PMC12356973; doi:10.1038/s41467-025-62832-5)

Supplementary information

Title: Iron-sulfur clusters in SARS-CoV-2 exoribonuclease and methyltransferase complexes: relevance for viral genome proofreading and capping

Authors: Nunziata Maio[1*], Umberto Terranova[2], Yan Li[3], J. Martin Bollinger Jr.[4,5], Carsten Krebs[4,5], and Tracey A. Rouault[1*]

Affiliations:

[1]*Eunice Kennedy Shriver* National Institute of Child Health and Human Development, National Institutes of Health, Bethesda, MD, 20892, USA

[2]Faculty of Medicine and Health Sciences, The University of Buckingham, Buckingham MK18 1EG & Crewe Campus, Crewe Green Road, Crewe CW1 5DU, UK

[3]National Institute of Neurological Disorders and Stroke, National Institutes of Health, Proteomics Core Facility, Bethesda, MD, 20892, USA

[4]Department of Chemistry, The Pennsylvania State University, University Park, PA, 16802, USA

[5]Department of Biochemistry and Molecular Biology, The Pennsylvania State University, University Park, PA, 16802, USA

***Correspondence to:** rouault@mail.nih.gov; nunziata.maio@nih.gov

Running title: Fe-S clusters in SARS-CoV-2 genome proofreading and capping

Keywords: exoribonuclease; methyltransferase complex; nsp14; nsp10; nsp16; iron-sulfur clusters

Table of Contents:

Supplementary Figure 1	2
Supplementary Figure 2	3
Supplementary Figure 3	4
Supplementary Figure 4	5
Supplementary Figure 5	6
List of mutagenesis primers	6
Supplementary References	7
Uncropped Gels and Blots	8

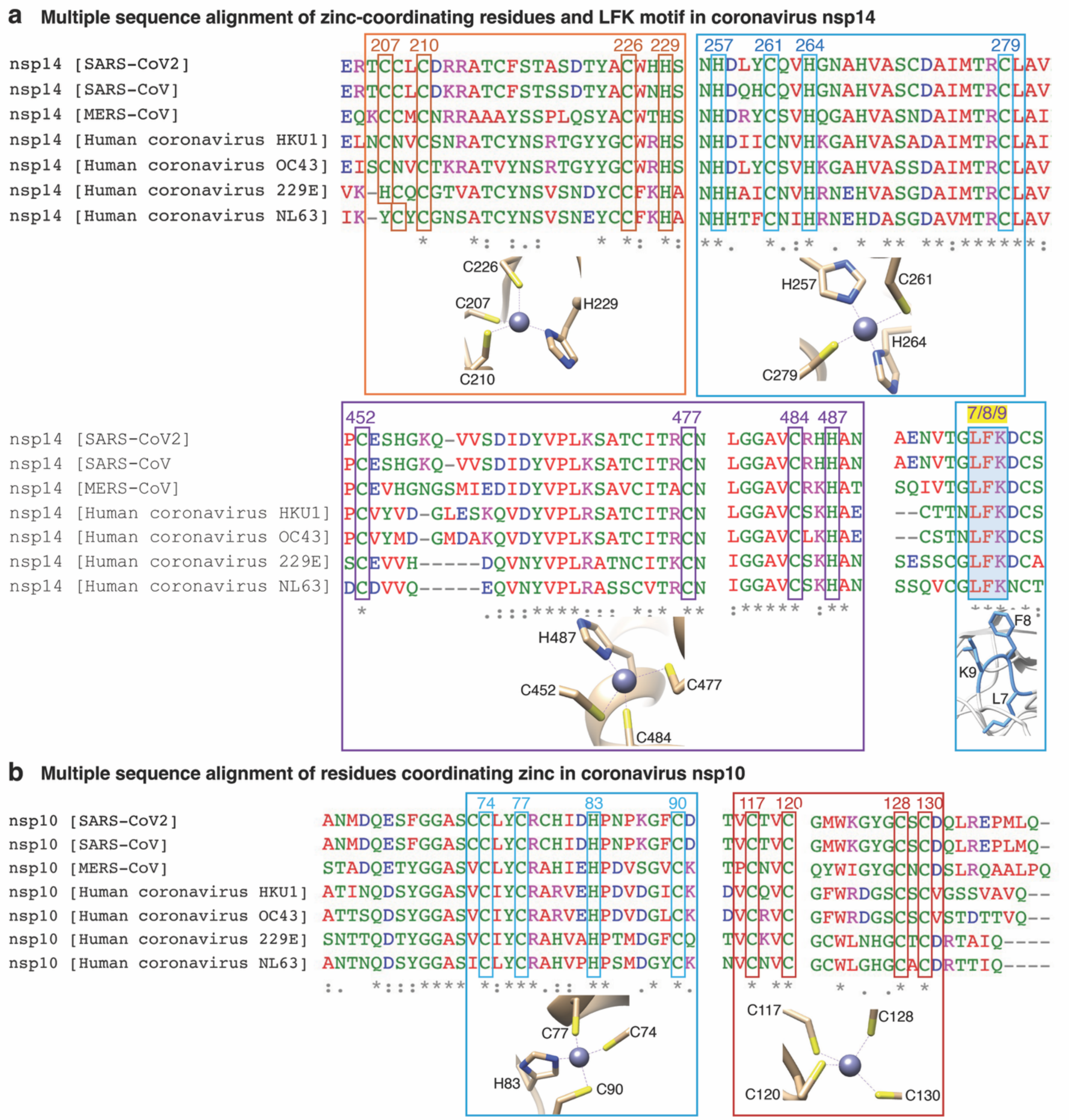

Supplementary Figure 1. Multiple sequence alignments of residues coordinating zinc in the available structures of the SARS-CoV-2 nsp14 and nsp10. a. Multiple sequence alignment of the residues ligating zinc and of the conserved LYR-like motif (Leu-Phe-Lys) in nsp14 of the seven human pathogenic coronaviruses. The LYR-like motif (LFK) is highlighted in yellow. Boxes in the same color identify the amino acid residues in each metal binding site. b. Multiple sequence alignment of the residues ligating zinc in nsp10 of the seven human pathogenic coronaviruses. Boxes in the same color identify the amino acid residues in each metal binding site.

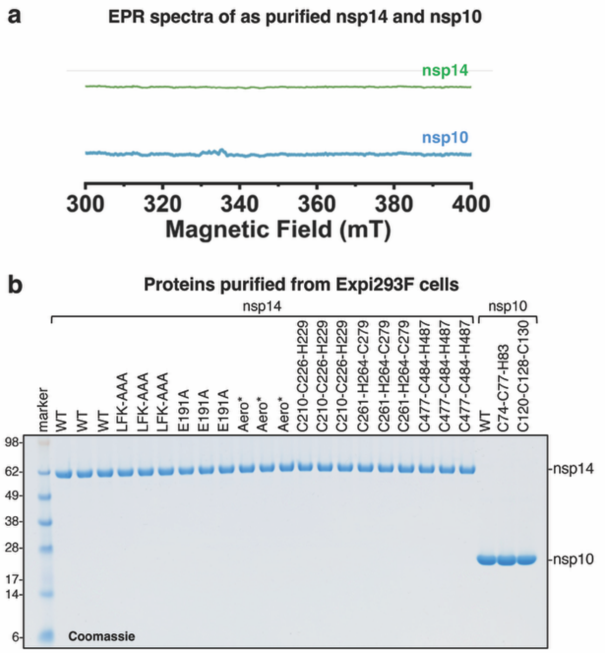

c Iron content in nsp14 and nsp10 WT and variants by ferrozine assay

nsp14:	Fe	SD
WT	7.5	1.9
LFK-AAA	0.3	0.1
E191A	7.2	1.0
C210-C226-H229	1.0	0.2
C261-H264-C279	0.2	0.2
C477-C484-H487	1.0	0.3
**nsp10:**		
WT	6.8	0.5
C74-C77-H83	0.5	0.4
C120-C128-C130	0.7	0.4

Supplementary Figure 2. EPR spectra of as-purified nsp14 and nsp10, Coomassie stain and iron content of purified wild-type and variant proteins. a. EPR spectra of as purified nsp14 and nsp10 recorded at 10K showing lack of signal ruling out the presence of Fe-S clusters with half integer spin ground state. b. Representative Coomassie staining of purified nsp14 and nsp10 WT and variant proteins analyzed by ICP-MS in Figures 2f and 2g (n=3 independent experiments). c. Iron content in nsp14 and nsp10 WT and variants as assessed by the colorimetric iron indicator, ferrozine (n=3 independent experiments). Source data are provided as a Source Data file and at the end of this document.

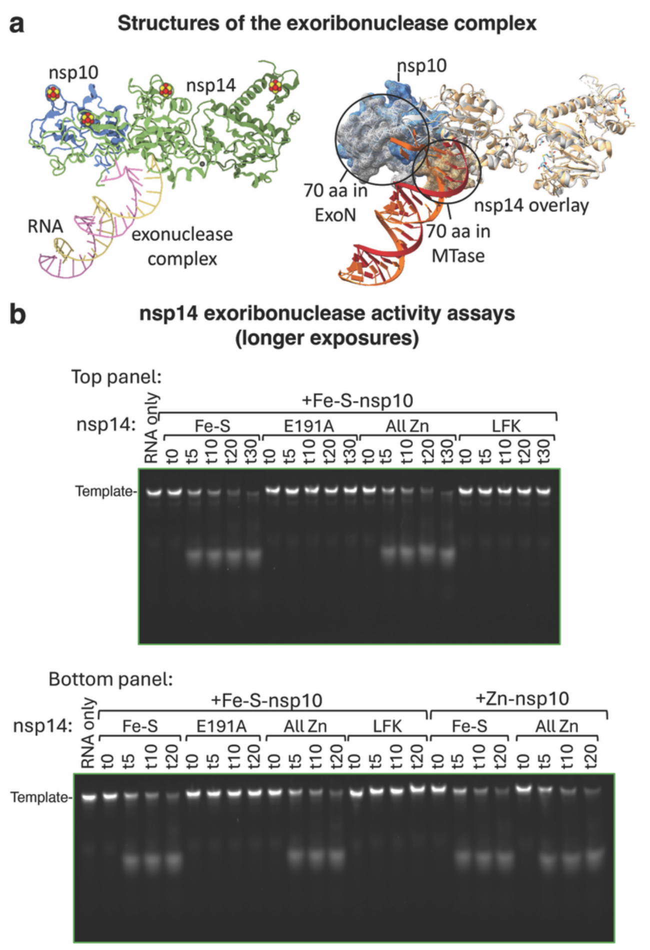

Supplementary Figure 3. Exoribonuclease complex and assay results of the ExoN activity: longer exposures of independent experiments. Related to Figure 3. a. Cartoon representation of the exoribonuclease complex, consisting of nsp14/nsp10 (PDB ID: 7N0C[1]), highlighting the metal centers in nsp14 and nsp10 characterized as Fe-S cluster ligating sites in the present study (on the left). Overlay of ExoN (nsp14/nsp10 in complex with RNA; PDB ID: 7N0C[1]) and MTase (nsp14 alone; PDB ID: 7R2V[2] and 7QGI[3]) reveals a distinct conformation of the N-terminal 70 amino acid residues of nsp14 in the two structures (right-hand side). The N-terminal region (residues 1–70) acts as a "lid" that occludes the nsp10 binding site. Upon nsp10 binding, the lid undergoes substantial refolding, forming a novel interaction surface that facilitates complex assembly and provides a mechanistic explanation for the allosteric regulation of nsp14 exonuclease activity by nsp10. b. ExoN activity assay of the nsp14/nsp10 complex monitored from time 0 (t0) to 30 minutes (t30). The assay was performed with nsp14 WT ligating either two Fe-S clusters (Fe-S nsp14) at the Cys_3His site or zinc at all three metal-binding sites (All Zn), in complex with nsp10 coordinating Fe-S clusters at its two metal-binding centers. Additionally, the assay included the catalytically inactive nsp14 E191A and an nsp14 variant in which the LFK motif was

replaced by triple alanines. Nsp14, either ligating two Fe-S clusters or zinc, was also tested in the presence of nsp10 ligating zinc (Zn-nsp10; lower panel) (n=3 independent experiments). Source data are provided as a Source Data file and at the end of this document.

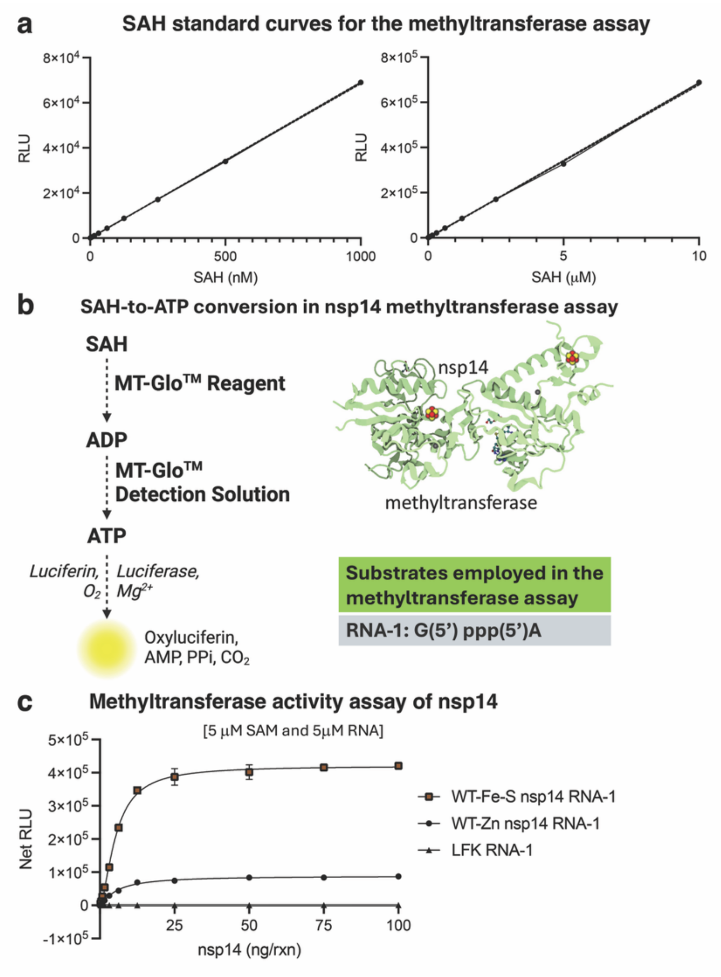

Supplementary Figure 4. Fe-S clusters in nsp14 specifically enhance methyltransferase activity without affecting exoribonuclease function. a. SAH standard curves for the methyltransferase activity measured from 0 to 1000 nM and from 0 to 10 μM. RLU = relative luminescence units. b. Schematic illustrating the conversion of S-adenosylhomocysteine (SAH) to ATP in the assay. Cartoon of nsp14 alone (PDB ID: 7R2V), highlighting the Fe-S clusters, is shown. The substrate used, RNA-1 (G(5')ppp(5')A), is indicated. c. Bioluminescence-based methyltransferase activity assay of nsp14 performed with increasing concentrations of WT nsp14, and LFK variant over 30 minutes, in the presence of 5 μM S-adenosylmethionine (SAM) and 5 μM RNA-1. Data are presented as mean values ± SD (n=3 independent experiments). Source data are provided as a Source Data file.

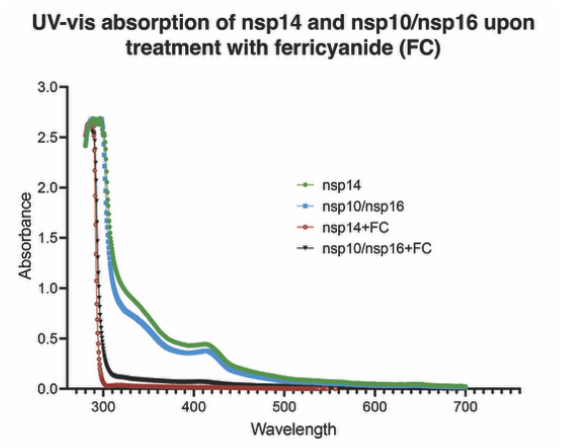

Supplementary Figure 5. Oxidation of Fe-S clusters in nsp14 or nsp10 by ferricyanide leads to cluster degradation, not a reversible 2+/3+ redox transition. UV-visible absorption spectra of purified nsp14 and the nsp10/nsp16 complex are shown before and after treatment with ferricyanide (1:1 molar ratio). Upon addition of ferricyanide, both proteins exhibit a loss of characteristic Fe-S absorbance features, consistent with cluster degradation rather than a stable $[Fe_4S_4]^{2+}/^{3+}$ redox transition. These results indicate that the Fe-S clusters are redox-active but structurally unstable upon oxidation under these conditions. Source data are provided as a Source Data file.

List of mutagenesis primers. List of mutagenesis primers used to generate the nsp14 and nsp10 variants reported in this study.

Primer name	Sequence
nsp14-LFK-AAA-F	5'-agtcctgtgatcactttactacagtctgcagccgcgcccgttacattttcagcagccgcagc-3'
nsp14-LFK-AAA-R	5'-gctgcggctgctgaaaatgtaacgggcgcggctgcagactgtagtaaagtgatcacaggact-3'
nsp14-E191A-F	5'-agtatttcatagaagtaagtgcgaagccatgtgcccacag-3'
nsp14-E191A-R	5'-ctgtgggcacatggcttcgcacttacttctatgaaatact-3'
nsp14-C210S-C226S-H229S-F	5'-aatcgaagcctatactgctgtgccagcttgcgtaggtgtcagatg-3'<br>5'-gagcggacatgttgtttgagcgaccgacgag-3'
nsp14-C210S-C226S-H229S-R	5'-catctgacacctacgcaagctggcacagcagtataggcttcgatt-3'<br>5'-ctcgtcggtcgctcaaacaacatgtccgctc-3'
nsp14-C261S-H264S-C279-F	5'-gtgcacggccaagctcctcgtcattatcg-3'<br>5'-cagagtaatcacgatctttacagccaagttagtgggaacgcacacg-3'
nsp14-C261S-H264S-C279-R	5'-cgataatgacgaggagcttggccgtgcac-3'<br>5'-cgtgtgcgttcccactaacttggctgtaaagatcgtgattactctg-3'
nsp14-C477S-C484S-H487S-F	5'-cgttggcactgtgtcggctaaccgcgcccccc-3'<br>5'-ccccccaaattgcttcgtgtaatgcacgttgca-3'
nsp14-C477S-C484S-H487S-R	5'-ggggggcgcggttagccgacacagtgccaacg-3'<br>5'-tgcaacgtgcattacacgaagcaatttgggggg-3'
nsp10-C74S-C77S-H83S-F	5'-cctttggggttaggactatctatatggcatctacagtacaaacag-3'<br>5'-ctatatggcatctactgtacaaactgcaactggcccctcc-3'

nsp10-C74S-C77S-H83S-R	5’-ctgtttgtactgtagatgccatatagatagtcctaaccccaaagg-3’<br>5’-ggaggggccagttgcagtttgtacagtagatgccatatag-3’
nsp10-C120S-C128S-C130S-F	5’-tctgagttggtcacttgagctgccgtaaccccttcc-3’<br>5’-cttccacatcccgctgacggtgcatacag-3’
nsp10-C120S-C128S-C130S-F	5’-ggaagggttacggcagctcaagtgaccaactcaga-3’<br>5’-ctgtatgcaccgtcagcgggatgtggaag-3’

Supplementary References

1 Liu, C. *et al*. Structural basis of mismatch recognition by a SARS-CoV-2 proofreading enzyme. *Science* **373**, 1142-1146 (2021). https://doi.org/10.1126/science.abi9310

2 Czarna, A. *et al*. Refolding of lid subdomain of SARS-CoV-2 nsp14 upon nsp10 interaction releases exonuclease activity. *Structure* **30**, 1050-1054 e1052 (2022). https://doi.org/10.1016/j.str.2022.04.014

3 Imprachim, N., Yosaatmadja, Y. & Newman, J. A. Crystal structures and fragment screening of SARS-CoV-2 NSP14 reveal details of exoribonuclease activation and mRNA capping and provide starting points for antiviral drug development. *Nucleic Acids Res* **51**, 475-487 (2023). https://doi.org/10.1093/nar/gkac1207

Uncropped gels and blots

Figure 1

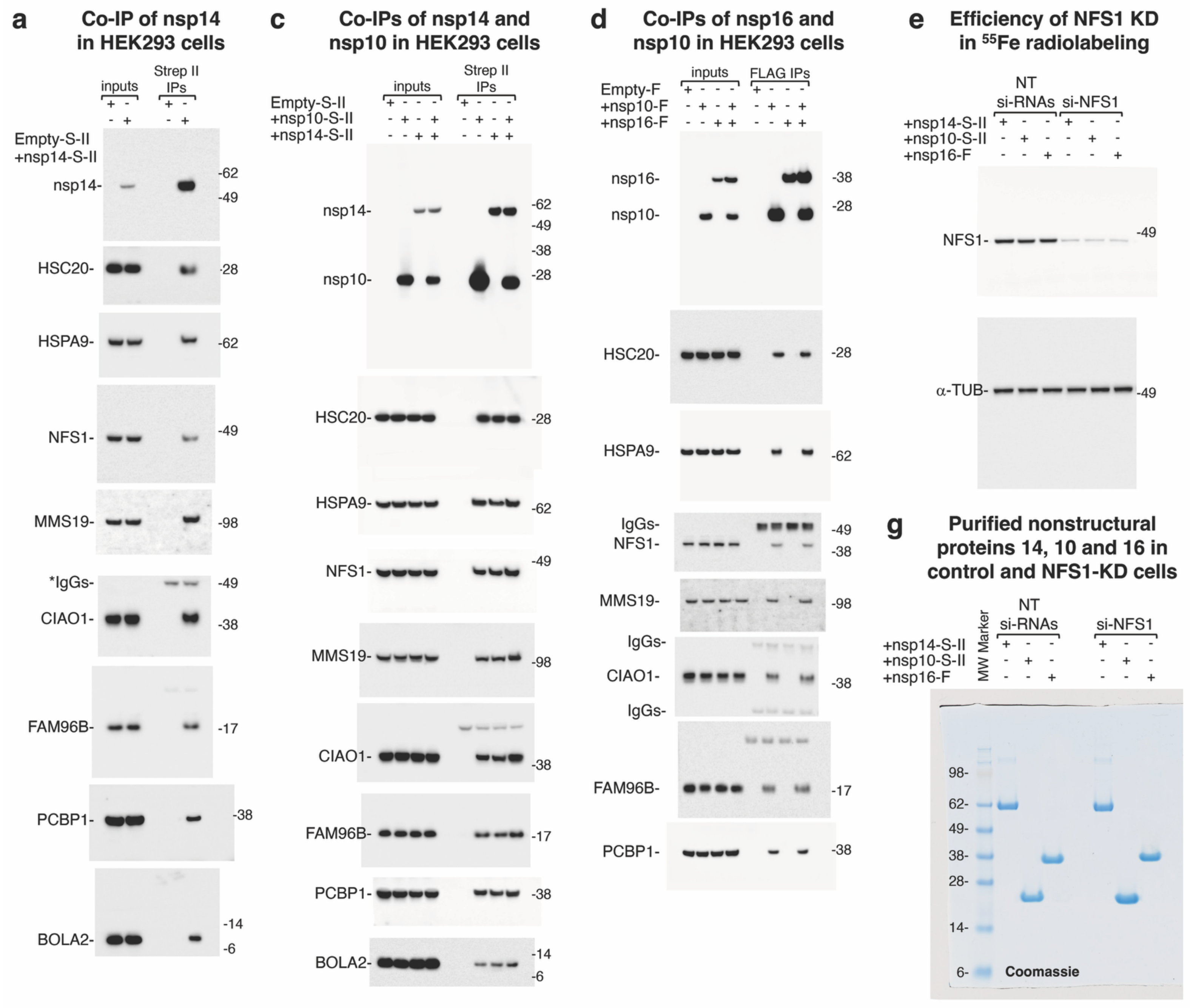

Figure 2

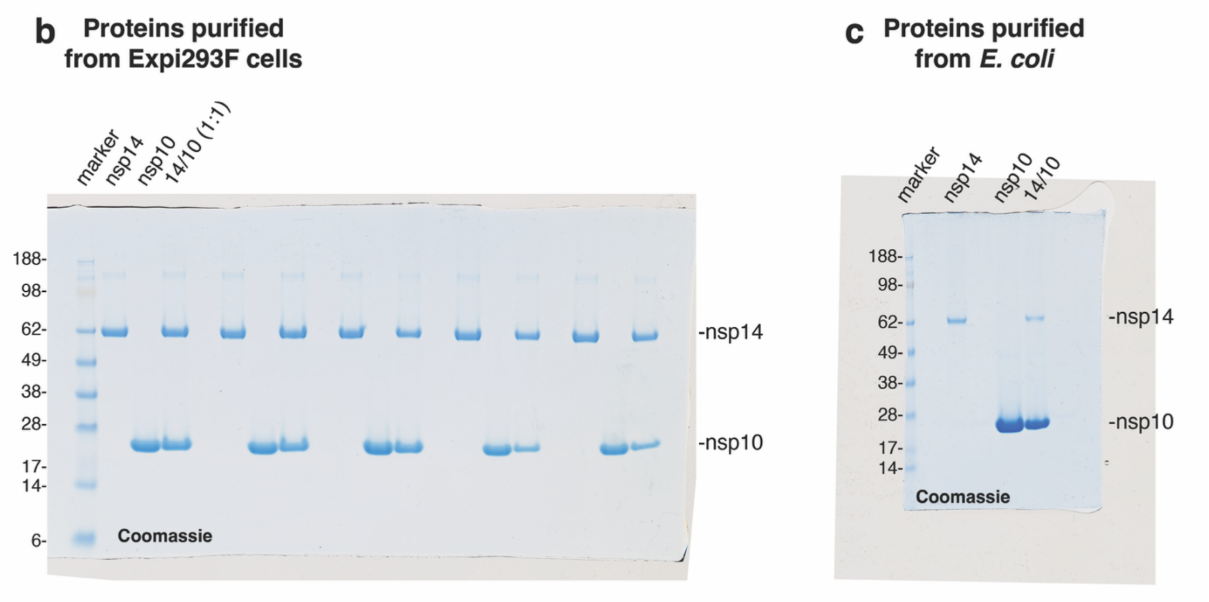
b Proteins purified from Expi293F cells
marker
nsp14
nsp10
14/10 (1:1)
188-
98-
62-
49-
38-
28-
17-
14-
6-
Coomassie
-nsp14
-nsp10
c Proteins purified from E. coli
marker
nsp14
nsp10
14/10
188-
98-
62-
49-
38-
28-
17-
14-
Coomassie
-nsp14
-nsp10

Figure 3

nsp14 exoribonuclease activity

Top panel (run at 100 V for 2h):

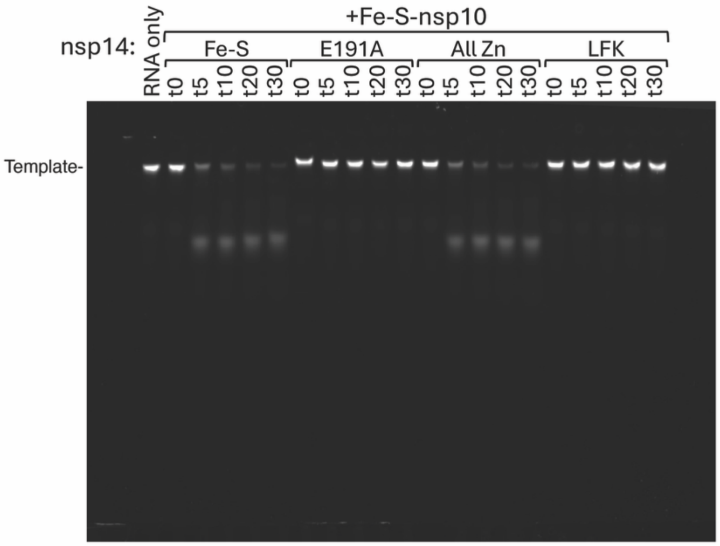

nsp14 exoribonuclease activity

Additional results not presented in main figures for Top panel (run at 100 V for 1h):

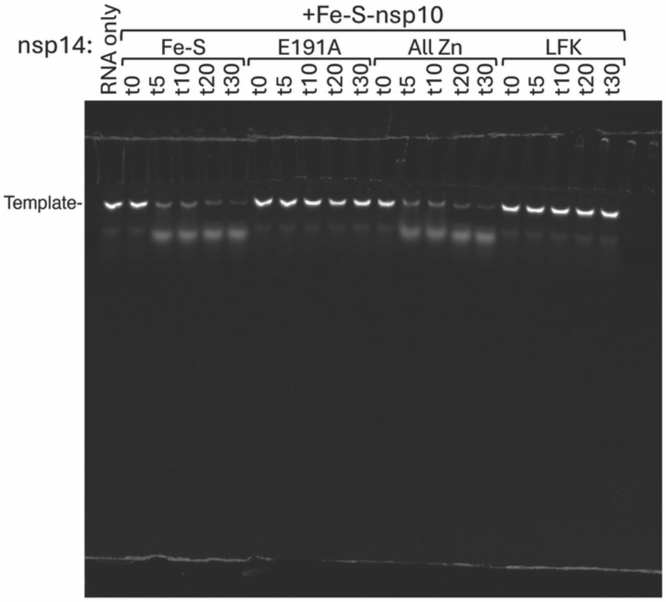

Bottom panel (run at 100 V for 2h):

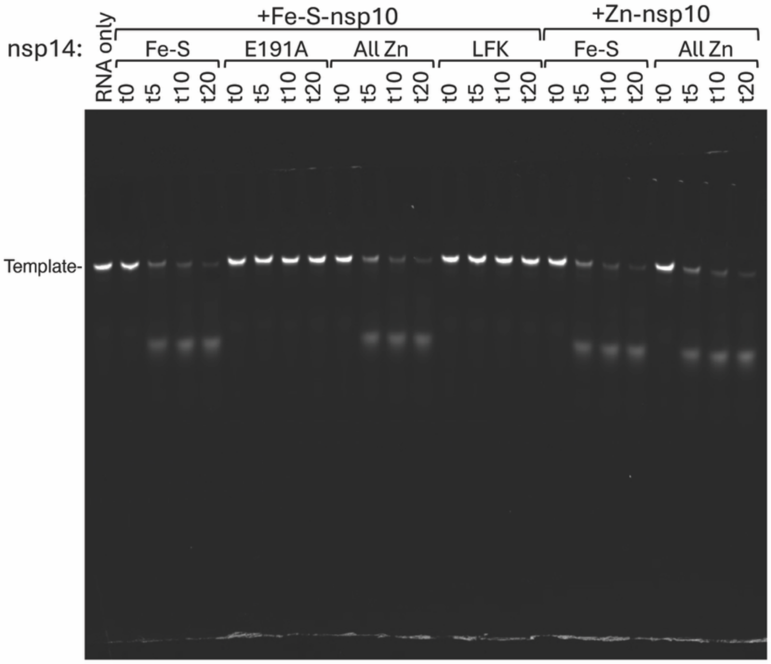

Additional results not presented in main figures for Bottom panel (run at 100 V for 1h):

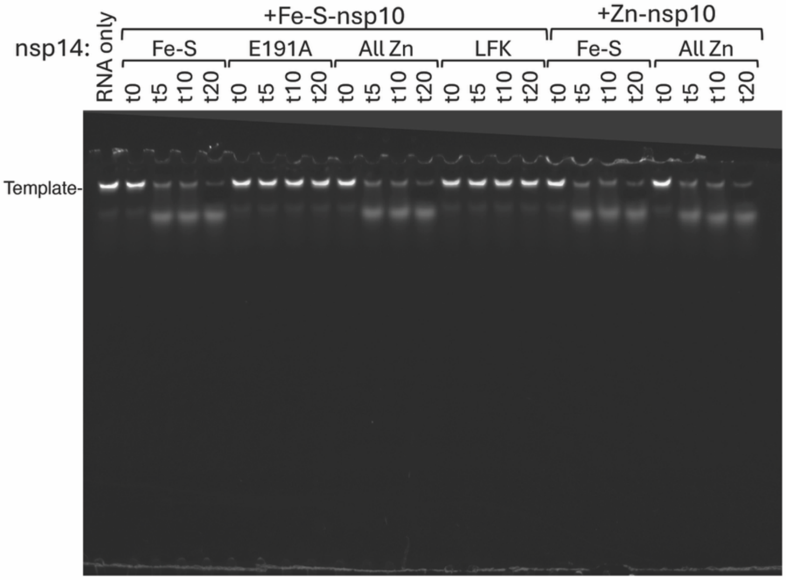

Figure 4

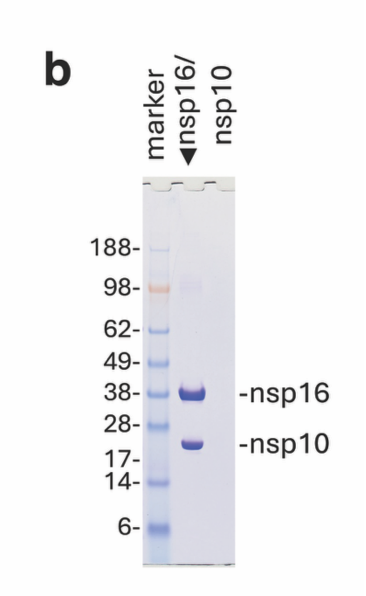

Figure 6

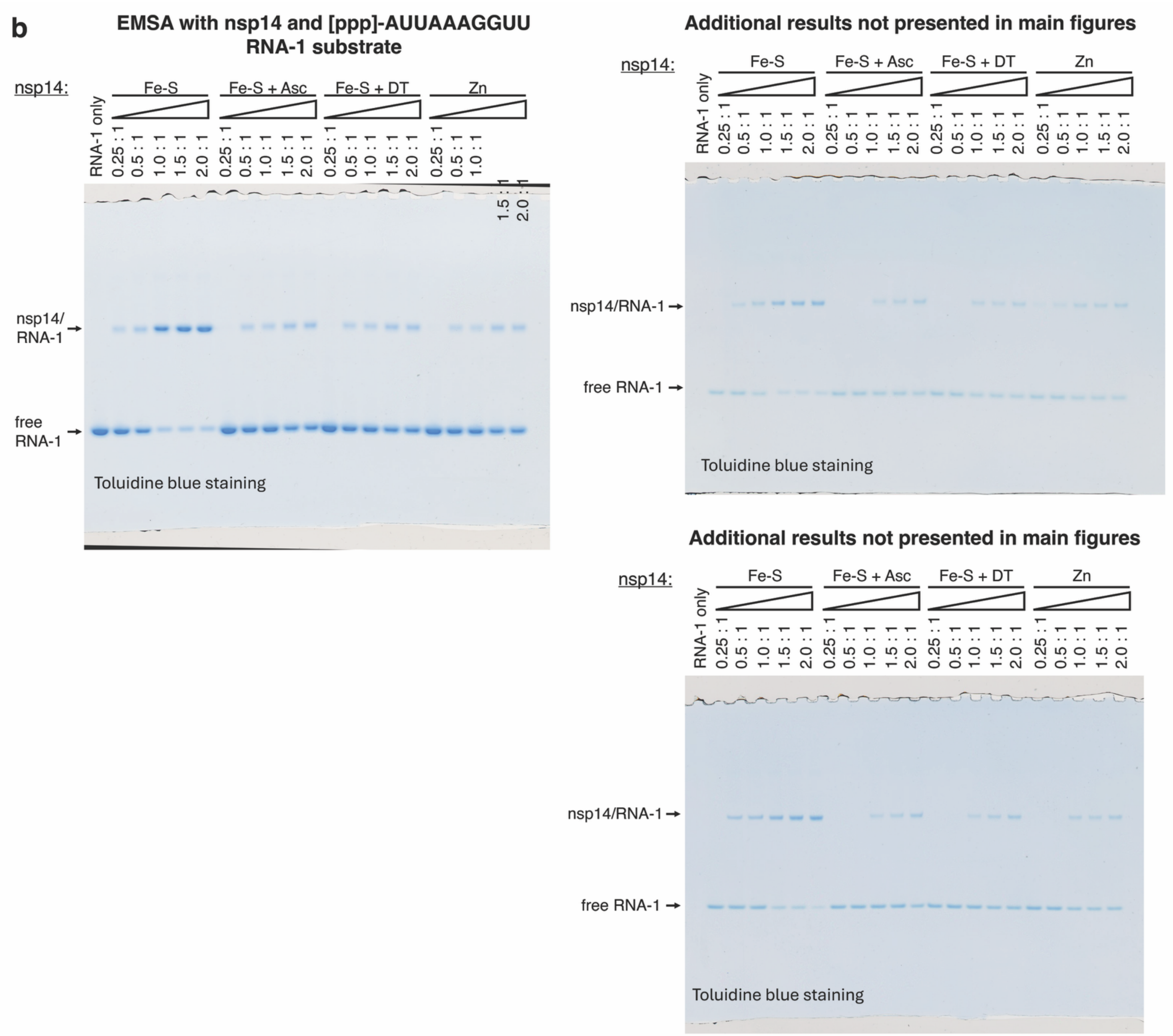
b
EMSA with nsp14 and [ppp]-AUUAAAGGUU RNA-1 substrate
nsp14:
Fe-S
Fe-S + Asc
Fe-S + DT
Zn
RNA-1 only
0.25 : 1
0.5 : 1
1.0 : 1
1.5 : 1
2.0 : 1
nsp14/ RNA-1
free RNA-1
Toluidine blue staining
Additional results not presented in main figures
nsp14:
Fe-S
Fe-S + Asc
Fe-S + DT
Zn
RNA-1 only
nsp14/RNA-1
free RNA-1
Toluidine blue staining
Additional results not presented in main figures
nsp14:
Fe-S
Fe-S + Asc
Fe-S + DT
Zn
RNA-1 only
nsp14/RNA-1
free RNA-1
Toluidine blue staining

Figure 6 (continued)

c Coomassie staining of nsp14 proteins

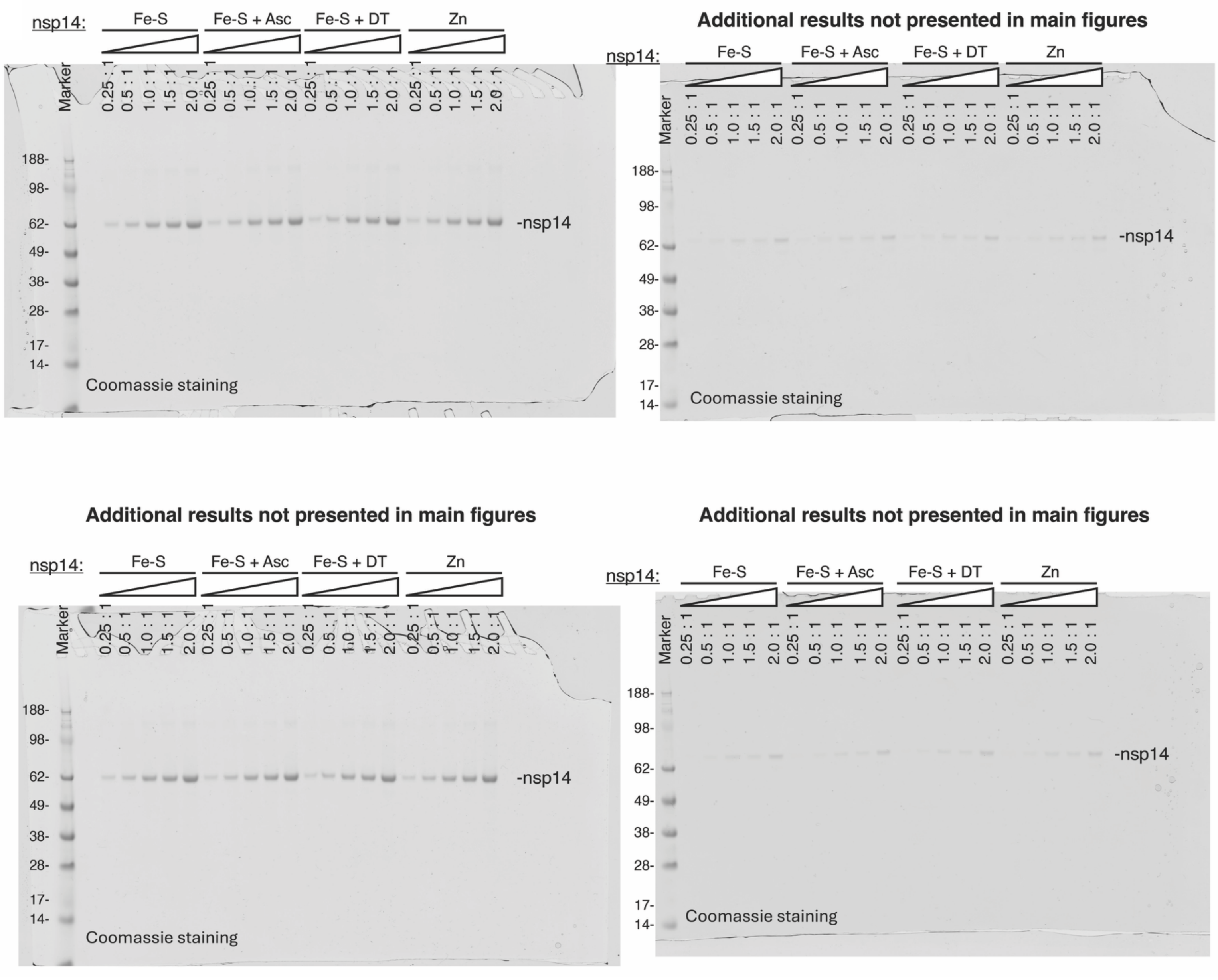

Figure 6 (continued)

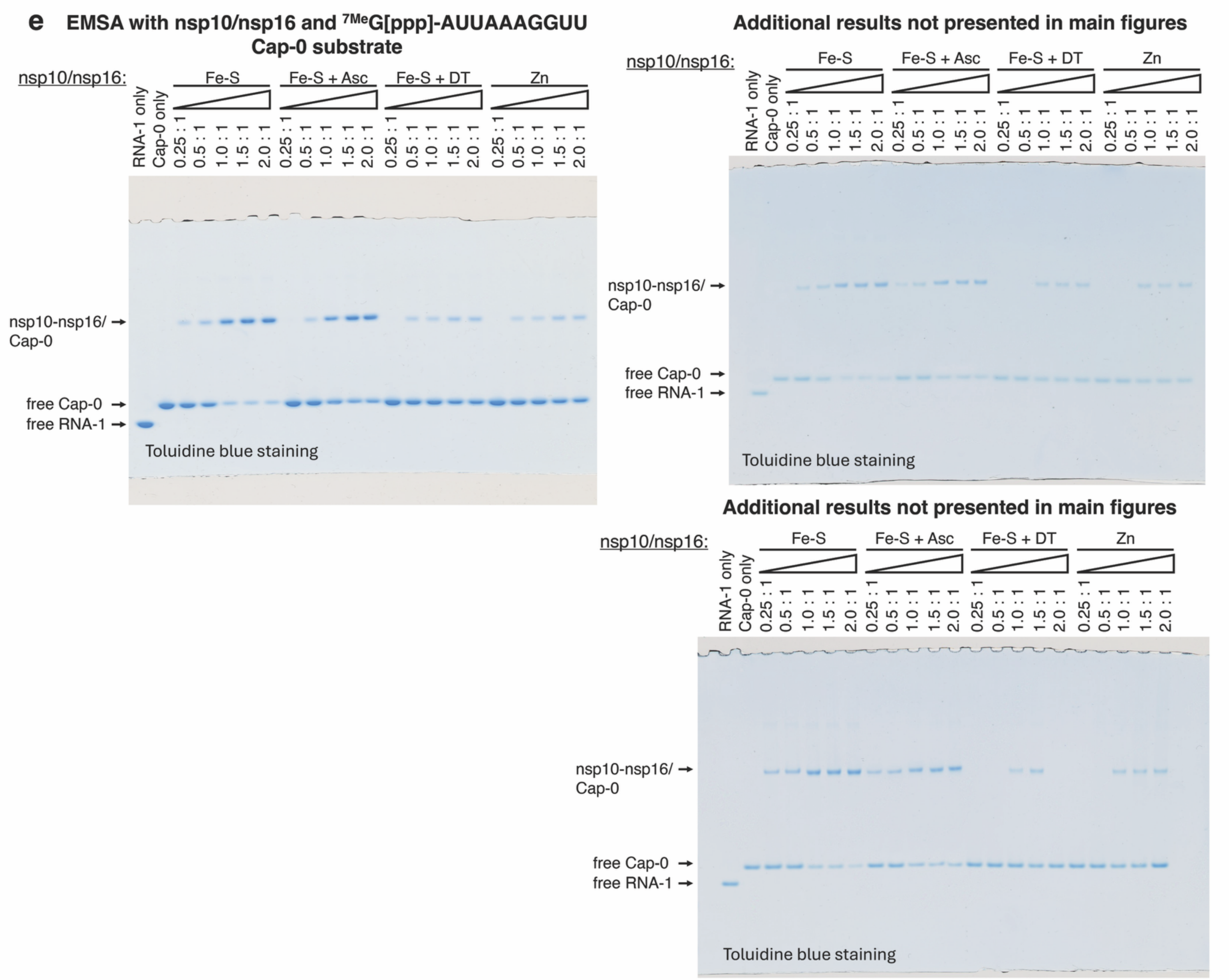

Figure 6 (continued)

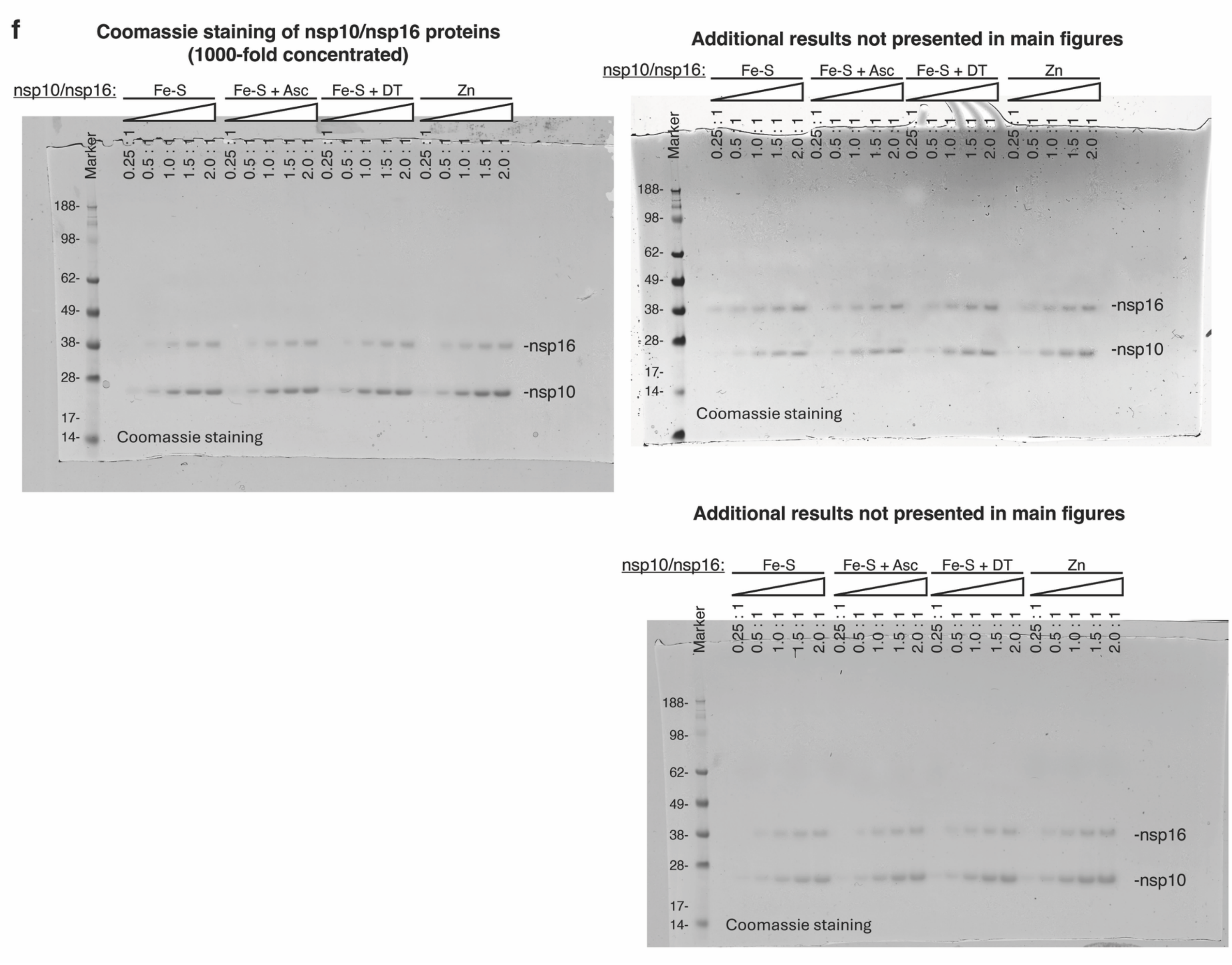

Supplementary Figure 2

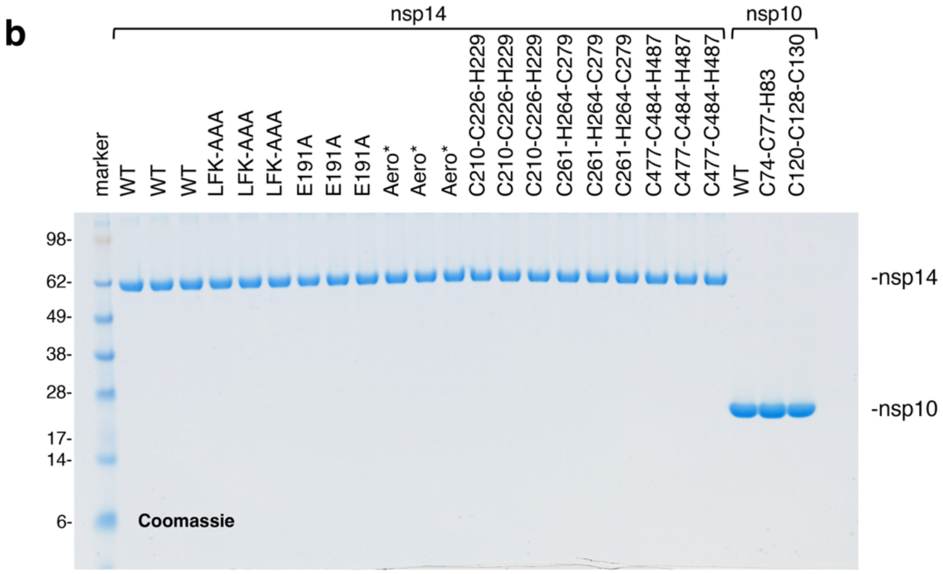

Supplementary Figure 3

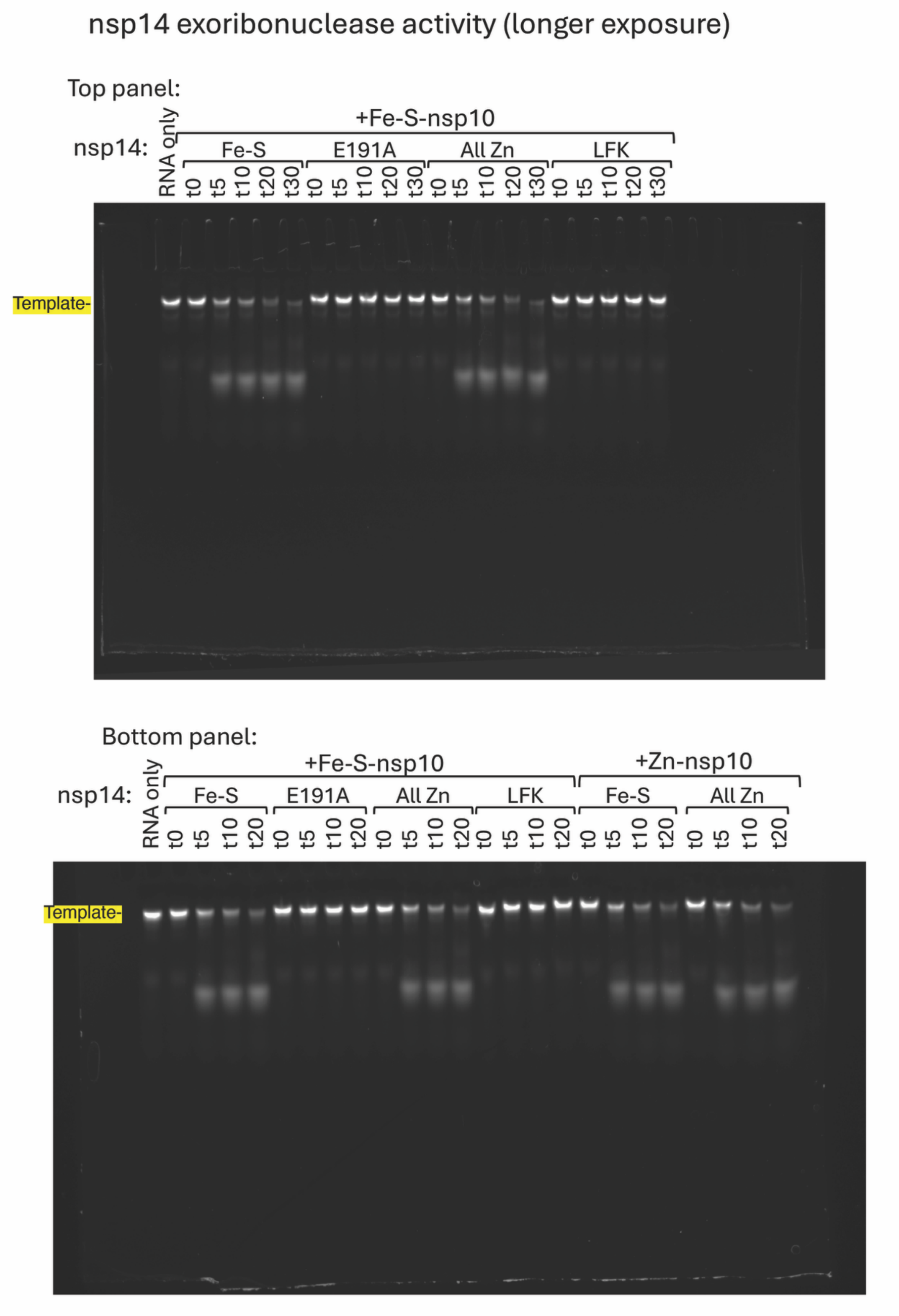

Supplement: Supplementary file 1 — Supplementary information [file 41467_2025_62832_MOESM1_ESM.pdf]
