# Supplementary material for: Iron-sulfur clusters in SARS-CoV-2 exoribonuclease and methyltransferase complexes: relevance for viral genome proofreading and capping
Source: Nat Commun. 2025 Aug 15;16:7585. doi: 10.1038/s41467-025-62832-5 (PMC12356973; doi:10.1038/s41467-025-62832-5)
Supplement: Supplementary file 2 — Description Of Additional Supplementary File [file 41467_2025_62832_MOESM2_ESM.pdf]

Description of Additional supplementary file

Supplementary data 1:

Full list of nsp14 interacting partners identified by mass spectrometry.
