# Supplementary material for: Iron-sulfur clusters in SARS-CoV-2 exoribonuclease and methyltransferase complexes: relevance for viral genome proofreading and capping
Source: Nat Commun. 2025 Aug 15;16:7585. doi: 10.1038/s41467-025-62832-5 (PMC12356973; doi:10.1038/s41467-025-62832-5)
Supplement: Supplementary file 4 — Reporting summary [file 41467_2025_62832_MOESM4_ESM.pdf]

nature portfolio

Corresponding author(s): Nunziata Maio, PhD nunziata.maio@nih.gov
Tracey A. Rouault MD rouault@mail.nih.gov

Last updated by author(s): July 22 2025

Reporting Summary

Nature Portfolio wishes to improve the reproducibility of the work that we publish. This form provides structure for consistency and transparency in reporting. For further information on Nature Portfolio policies, see our Editorial Policies and the Editorial Policy Checklist.

Statistics

For all statistical analyses, confirm that the following items are present in the figure legend, table legend, main text, or Methods section.

n/a	Confirmed	
☐	☒	The exact sample size (*n*) for each experimental group/condition, given as a discrete number and unit of measurement
☐	☒	A statement on whether measurements were taken from distinct samples or whether the same sample was measured repeatedly
☐	☒	The statistical test(s) used AND whether they are one- or two-sided<br>*Only common tests should be described solely by name; describe more complex techniques in the Methods section.*
☒	☐	A description of all covariates tested
☒	☐	A description of any assumptions or corrections, such as tests of normality and adjustment for multiple comparisons
☐	☒	A full description of the statistical parameters including central tendency (e.g. means) or other basic estimates (e.g. regression coefficient) AND variation (e.g. standard deviation) or associated estimates of uncertainty (e.g. confidence intervals)
☐	☒	For null hypothesis testing, the test statistic (e.g. *F*, *t*, *r*) with confidence intervals, effect sizes, degrees of freedom and *P* value noted<br>*Give P values as exact values whenever suitable.*
☒	☐	For Bayesian analysis, information on the choice of priors and Markov chain Monte Carlo settings
☒	☐	For hierarchical and complex designs, identification of the appropriate level for tests and full reporting of outcomes
☒	☐	Estimates of effect sizes (e.g. Cohen's *d*, Pearson's *r*), indicating how they were calculated

Our web collection on statistics for biologists contains articles on many of the points above.

Software and code

Policy information about availability of computer code

Data collection	Proteome Discoverer (version 2.4) - for LC-MS/MS data procressing and protein identification; Bruker's Xepr software - acquisition software for Electron Paramagnetic Resonance spectra.
Data analysis	VMD (Visual Molecular Dynamics); NAMD – for molecular dynamics (MD) simulations; CP2K – for quantum mechanics/molecular mechanics (QM/MM) calculations and density functional theory (DFT)-based energy evaluations; CHARMM36 force field – For modeling molecular mechanics interactions; Bio-Rad Chemidoc™ Image Lab Software – for gel image analysis; GraphPad Prism (version 10.2.3) – for statistical analysis, including two-way ANOVA and multiple comparisons; EasySpin package 6.0.6 was used to simulate the EPR spectra.

For manuscripts utilizing custom algorithms or software that are central to the research but not yet described in published literature, software must be made available to editors and reviewers. We strongly encourage code deposition in a community repository (e.g. GitHub). See the Nature Portfolio guidelines for submitting code & software for further information.

Data

Policy information about availability of data

All manuscripts must include a data availability statement. This statement should provide the following information, where applicable:

- Accession codes, unique identifiers, or web links for publicly available datasets
- A description of any restrictions on data availability
- For clinical datasets or third party data, please ensure that the statement adheres to our policy

All data needed to evaluate the conclusions of the paper are present in the main text and supplementary information. Source data are provided with this paper. The mass spectrometry data have been deposited to ProteomeXchange PXD060605 (https://proteomecentral.proteomexchange.org/cgi/GetDataset?ID=PXD060605).

Research involving human participants, their data, or biological material

Policy information about studies with human participants or human data. See also policy information about sex, gender (identity/presentation), and sexual orientation and race, ethnicity and racism.

Reporting on sex and gender	No human participants or human data involved in this study.
Reporting on race, ethnicity, or other socially relevant groupings	No human participants or human data involved in this study.
Population characteristics	No human participants or human data involved in this study.
Recruitment	No human participants or human data involved in this study.
Ethics oversight	No human participants or human data involved in this study.

Note that full information on the approval of the study protocol must also be provided in the manuscript.

Field-specific reporting

Please select the one below that is the best fit for your research. If you are not sure, read the appropriate sections before making your selection.

☒ Life sciences ☐ Behavioural & social sciences ☐ Ecological, evolutionary & environmental sciences

For a reference copy of the document with all sections, see nature.com/documents/nr-reporting-summary-flat.pdf

Life sciences study design

All studies must disclose on these points even when the disclosure is negative.

Sample size

No formal statistical methods were used to predetermine sample sizes for the experiments in this study. Sample sizes were chosen based on standard practices in biochemical and biophysical characterization of proteins and their cofactors. For key experiments:
Iron incorporation assays: Typically performed in quadruplicates (n = 4 biological replicates), providing sufficient replication to assess consistency while balancing the use of radioisotopes.
Enzymatic activity assays: Sample sizes were determined based on the need to obtain reliable kinetic data. Multiple time points (0-30 minutes) were used for exonuclease assays, and reactions were typically performed in triplicate to ensure reproducibility.
Spectroscopic analyses: Sample sizes were dictated by the requirements of each technique (e.g., ICP-MS, UV-visible absorption, EPR spectroscopy) to obtain clear and interpretable spectra (n = 3 biological replicates).
The chosen sample sizes were sufficient to demonstrate clear and reproducible effects, as evidenced by the consistent results obtained across multiple experimental approaches. These sample sizes are in line with those commonly used in similar biochemical studies characterizing protein cofactors and enzymatic activities.
For cellular and biochemical assays, a minimum of three independent biological replicates was used to ensure reproducibility of the results. The statistical analyses, including two-way ANOVA and Sidak's multiple comparisons test, were applied to assess the significance of the differences between groups, with data expressed as mean ± SD.

Data exclusions

No data were excluded from the analysis.

Replication

Reproducibility and Replication
All experimental findings reported in this study were successfully replicated and verified for reproducibility. Specific measures taken to ensure reproducibility include:
Multiple independent experiments: Experiments, such as iron incorporation assays, enzymatic activity assays, and spectroscopic analyses, were performed at least three times independently to confirm consistency of results.
Technical replicates: Within each independent experiment, technical replicates (typically triplicates) were used to assess intra-experimental variability and ensure consistency.

Diverse methodologies: The use of complementary techniques (e.g., ICP-MS, UV-visible absorption, EPR spectroscopy, and radioactive iron incorporation assays) provided multiple lines of evidence supporting our conclusions about the presence and function of Fe-S clusters in nsp14 and nsp10.
Controls: Appropriate controls were included in all experiments, such as background measurements in iron incorporation assays and the use of non-targeting siRNAs in knockdown experiments.
Standardized protocols: Detailed protocols were established and followed consistently across all replications to minimize experimental variability.
Data validation: Raw data from each experiment were carefully analyzed and cross-checked by multiple team members to ensure accurate interpretation and reproducibility of findings.
All attempts at replication were successful, and no findings failed to be reproduced. The consistency across multiple experimental approaches and replicates strengthens the reliability of our conclusions.

Randomization

The nature of this biochemical study did not require randomization or allocation procedures typically associated with clinical trials or animal studies. Our experiments involved:
Protein purification and characterization
In vitro enzymatic assays
Spectroscopic analyses
These methodologies are based on controlled biochemical reactions and physical measurements, where the concept of randomization is not applicable.
Experimental controls: Instead of randomization, we ensured experimental rigor through:
Use of appropriate positive and negative controls in each experiment
Multiple independent replicates of key experiments
Standardized protocols for protein preparation and assay conditions
Addressing potential bias: To minimize potential sources of bias:
Samples were prepared and analyzed in a consistent manner across all experiments
Measurements were taken using calibrated instruments
Data analysis followed pre-established protocols
This approach ensured that our results were reproducible and representative of the true biochemical properties of the proteins and cofactors under study.

Blinding

Blinding
Due to the nature of the experiments conducted in this study, blinding was not possible.
Justification:
Our research primarily involves in vitro biochemical and biophysical characterization of purified proteins. Specifically:
Enzyme activity assays: The assays involve direct measurement of enzyme activity with defined substrates and products. The readout is quantitative (e.g., luminescence, gel electrophoresis), and the experimenter directly observes the changes in substrate/product concentrations.
Spectroscopic analyses: Similarly, in spectroscopic experiments (UV-Vis, EPR, ICP-MS), the measurements are direct physical characterizations of the purified proteins or cofactors. The data is objectively acquired and interpreted.
Iron incorporation assays: These experiments quantify the incorporation of radioactive iron into purified proteins. The process involves direct measurement using scintillation counting.
In all of these methodologies, the researcher is directly involved in setting up the reactions, collecting the data, and interpreting the results. The assays are not amenable to blinding because the experimenter is fully aware of the sample identity and experimental conditions throughout the process. This is a common limitation in basic biochemical and biophysical studies. However, we mitigated potential bias by:
Including appropriate controls in all experiments
Performing experiments in replicates and independently
Adhering to standardized, well-defined protocols
Cross-validating results with multiple, complementary techniques
This approach ensures the objectivity and reliability of our findings, despite the absence of blinding.

Reporting for specific materials, systems and methods

We require information from authors about some types of materials, experimental systems and methods used in many studies. Here, indicate whether each material, system or method listed is relevant to your study. If you are not sure if a list item applies to your research, read the appropriate section before selecting a response.

Materials & experimental systems

n/a	Involved in the study
☐	☒ Antibodies
☐	☒ Eukaryotic cell lines
☒	☐ Palaeontology and archaeology
☒	☐ Animals and other organisms
☒	☐ Clinical data
☒	☐ Dual use research of concern
☒	☐ Plants

Methods

n/a	Involved in the study
☒	☐ ChIP-seq
☒	☐ Flow cytometry
☒	☐ MRI-based neuroimaging

Antibodies

Antibodies used	Antibodies<br>Antibodies in this study were as follows: anti-HSC20 westerns were performed either with a custom-made antibody raised against the whole protein (Genscript) or with a commercial antibody (Sigma #HPA018447). Anti-CIAO1 (sc-374498) and NFS1 (sc-81107) were from Santa Cruz Biotechnology. Anti-HSPA9 (HPA000898) was from Sigma. Anti-FAM96B (20108-1-AP) and MMS19 (16015-1-AP) were from Proteintech. Anti-FLAG antibody was from Origene (TA50011). Anti-Strep II (ab184224) was from Qiagen (1023944). Anti-PCBP1 was from Santa Cruz Biotechnology (sc-393075). Anti-BOLA2 was from Bethyl Laboratories (A305-890A-M). Anti alpha-Tubulin was obtained from Sigma (T9026). Primary antibodies were used at a 1:1,000 dilution and incubated overnight at 4°C.
Validation	The antibodies used in this study have been extensively validated in previous publications [PMIDs: 38950322; 37552760; 34083449; 29309586]. Validation methods have included:<br>Prior publications: The antibodies have a proven track record of specificity and reliability in previously published studies (e.g., those listed above).<br>Knockdown validation: Antibody specificity has often been confirmed by siRNA-mediated knockdown experiments. In these experiments, the target protein is depleted using siRNA, and the corresponding antibody signal is shown to be significantly reduced or eliminated. This confirms that the antibody specifically recognizes the intended target and does not exhibit significant off-target binding [PMIDs: 38950322; 37552760; 34083449; 29309586].<br>Immunoprecipitation followed by Mass Spectrometry: The proteins of interest were immunoprecipitated using specific antibodies and the identities were confirmed by mass spectrometry.<br>Western blotting: Standard western blotting procedures were used to confirm the presence of the protein using the validated antibodies.<br>This multi-pronged approach to antibody validation, combining prior publication, knockdown validation, and orthogonal methods, ensures the reliability and specificity of the antibodies used in this study.

Eukaryotic cell lines

Policy information about cell lines and Sex and Gender in Research

Cell line source(s)	HEK293 cells were purchased from ATCC (#CRL-1573™). Cells were propagated in Dulbecco's modified Eagle's medium (DMEM) with 4.5g/L glucose, supplemented with 10% fetal bovine serum (FBS) and 2mM glutamine at 37 C and 5% CO_2 in a humidified incubator. Expi293F cells used for mammalian cell expression of the SARS-CoV-2 nsp14, nsp10 and nsp16 were purchased from ThermoFisher Scientific (#A14635). Cells were propagated in suspension in chemically defined, serum-free, protein-free, animal origin-free Expi293 Expression Medium at 37 C and 8% CO_2 and subcultured according to the manufacturer's instructions. All cell lines were subjected to mycoplasma testing.
Authentication	Cell Line Authentication<br>The Expi293F cells were obtained directly from ThermoFisher Scientific and HEK293 cells from ATCC. While we did not perform authentication in this study, we implemented the following measures to ensure cell line identity and integrity:<br>Traceability: We maintained careful records of the cell lines passage history and handling.<br>Consistent phenotype: We monitored the cells regularly for expected morphology and growth characteristics.<br>Early passage use: Cells were used at low passage numbers to minimize the risk of genetic drift or contamination.<br>Mycoplasma testing: As stated previously, all cell lines used in this study underwent routine testing for mycoplasma contamination, ensuring that any observed effects were not due to mycoplasma infection.<br>The measures taken provide reasonable assurance of the identity and stability of the cell lines used in this study.
Mycoplasma contamination	All cell lines in this study tested negative for mycoplasma.
Commonly misidentified lines<br>(See ICLAC register)	N/A.

Plants

Seed stocks	No plants involved in this study.
Novel plant genotypes	No plants involved in this study.
Authentication	No plants involved in this study.
